# Generation of KCL017 research grade human embryonic stem cell line carrying a mutation in VHL gene

**DOI:** 10.1016/j.scr.2016.01.013

**Published:** 2016-03

**Authors:** Heema Hewitson, Victoria Wood, Neli Kadeva, Glenda Cornwell, Stefano Codognotto, Emma Stephenson, Dusko Ilic

**Affiliations:** Stem Cell Laboratories, Division of Women's Health, Faculty of Life Sciences and Medicine, King's College London and Assisted Conception Unit, Guys' Hospital, London, United Kingdom

## Abstract

The KCL017 human embryonic stem cell line was derived from an embryo donated for research that carried an autosomal dominant mutation affecting splicing site of the VHL gene encoding von Hippel–Lindau tumor suppressor E3 ubiquitin protein ligase (676 + 3 A > T). The ICM was isolated using laser microsurgery and plated on γ-irradiated human foreskin fibroblasts. Both the derivation and cell line propagation were performed in an animal product-free environment. Pluripotent state and differentiation potential were confirmed by in vitro assays.

## Resource table

**Table:** 

Name of stem cell line	KCL017
Institution	King's College London, London UK
Derivation team	Neli Kadeva, Victoria Wood, Glenda Cornwell, Stefano Codognotto, Emma Stephenson
Contact person and email	Dusko Ilic, email: dusko.ilic@kcl.ac.uk
Type of resource	Biological reagent: cell line
Sub-type	Human pluripotent stem cell line
Origin	Human embryo
Key marker expression	Pluripotent stem cell markers: NANOG, OCT4, TRA-1-60, TRA-1-81, alkaline phosphatase (AP) activity
Authentication	Identity and purity of line confirmed
Link to related literature (direct URL links and full references)	1) Ilic, D., Stephenson, E., Wood, V., Jacquet, L., Stevenson, D., Petrova, A., Kadeva, N., Codognotto, S., Patel, H., Semple, M., Cornwell, G., Ogilvie, C., Braude, P., 2012. Derivation and feeder-free propagation of human embryonic stem cells under xeno-free conditions. Cytotherapy. 14 (1), 122–128. doi: 10.3109/14,653,249.2011.623692 http://www.ncbi.nlm.nih.gov/pubmed/22029654 2) Stephenson, E., Jacquet, L., Miere, C., Wood, V., Kadeva, N., Cornwell, G., Codognotto, S., Dajani, Y., Braude, P., Ilic, D., 2012. Derivation and propagation of human embryonic stem cell lines from frozen embryos in an animal product-free environment. Nat. Protoc. 7 (7), 1366–1381. doi: 10.1038/nprot.2012.080 http://www.ncbi.nlm.nih.gov/pubmed/22722371
Information in public databases	KCL017 is a National Institutes of Health (NIH) registered hESC line NIH Registration Number: 0217 NIH Approval Number: NIHhESC-13-0217 http://grants.nih.gov/stem_cells/registry/current.htm?id=657
Ethics	The hESC line KCL017 is derived under license from the UK Human Fertilisation and Embryology Authority (research license numbers: R0075 and R0133) and also has local ethical approval (UK National Health Service Research Ethics Committee Reference: 06/Q0702/90). Informed consent was obtained from all subjects and the experiments conformed to the principles set out in the WMA Declaration of Helsinki and the NIH Belmont Report. No financial inducements are offered for donation.

## Resource details

**Table:** 

Consent signed	Aug 12, 2009
Embryo used	Aug 23, 2009
UK Stem Cell Bank Deposit Approval	Sep 23, 2010 Reference: SCSC10-35
Sex	Male 46, XY
Grade	Research
Disease status (Fig 1)	Mutation affecting splicing site of the VHL gene encoding von Hippel–Lindau tumor suppressor E3 ubiquitin protein ligase (676 + 3 A > T)
Karyotype (aCGH)	No imbalance detected
DNA fingerprint (Table 1)	Allele sizes (in bp) of 16 microsatellite markers specific for chromosomes 13, 18 and 21
Viability testing	Pass
Pluripotent markers (immunostaining) (Fig 2)	NANOG, OCT4, TRA-1-60, TRA-1-81, AP activity
Three germ layers differentiation in vitro (immunostaining) (Fig 3)	Endoderm: AFP (α-fetoprotein) Ectoderm: TUBB3 (tubulin, β3 class III) Mesoderm: ACTA2 (actin, α2, smooth muscle)
Sibling lines available	KCL015, KCL016

We generated KCL017 clinical grade hESC line following protocols, established previously (Ilic et al., 2012, Stephenson et al., 2012). The expression of the pluripotency markers was tested after freeze/thaw cycle. Differentiation potential into three germ layers was verified in vitro.

## Materials and methods

### Consenting process

We distribute Patient Information Sheet (PIS) and consent form to the in vitro fertilization (IVF) patients if they opted to donate to research embryos that were stored for 5 or 10 years. They mail signed consent back to us and that might be months after the PIS and consent were mailed to them. If in the meantime new versions of PIS/consent are implemented, we do not send these to the patients or ask them to re-sign; the whole process is done with the version that was given them initially. The PIS/consent documents (PGD-V.6) were created on Aug. 10, 2007. HFEA Code of Practice that was in effect at the time of document creation: Edition 7 — R.1 (http://www.hfea.gov.uk/2999.html). The donor couple signed the consent on Aug. 12, 2009. HFEA Code of Practice that was in effect at the time of donor signature: Edition 7 — R.4. HFEA Code of Practice Edition 7 – R.1 was in effect until 09 Dec. 2007 and Edition 7 — R.4 was in effect: 02 Oct. 2008–30 Sep. 2009.

### Embryo culture and micromanipulation

Embryo culture and laser-assisted dissection of inner cell mass (ICM) were carried out as previously described in details (Ilic et al., 2012, Stephenson et al., 2012). The cellular area containing the ICM was then washed and transferred to plates containing mitotically inactivated human neonatal foreskin fibroblasts (HFF).

### Cell culture

ICM plated on mitotically inactivated HFF were cultured as described (Ilic et al., 2012, Stephenson et al., 2012). TE cells were removed mechanically from outgrowth (Ilic et al., 2007, Ilic et al., 2010). hES colonies were expanded and cryopreserved at the third passage.

### Viability test

Straws with the earliest frozen passage (p.2–3) are thawed and new colonies are counted three days later. These colonies are then expanded up to passage 8, at which point cells were part frozen and part subjected to standard battery of tests (pluripotency markers, in vitro and in vivo differentiation capability, genetics, sterility, and mycoplasma).

### Pluripotency markers

Pluripotency was assessed using two different techniques: enzymatic activity assay [alkaline phosphatase (AP) assay] and immunostaining as described (Ilic et al., 2012, Stephenson et al., 2012, Petrova et al., 2014).

### Differentiation

Spontaneous differentiation into three germ layers was assessed in vitro as described (Ilic et al., 2012, Stephenson et al., 2012, Petrova et al., 2014).

### Genotyping

DNA was extracted from hES cell cultures using a Chemagen DNA extraction robot according to the manufacturer's instructions. Amplification of polymorphic microsatellite markers was carried out as described (Ilic et al., 2012). Allele sizes were recorded to give a unique fingerprint of each cell line.

### Array comparative genomic hybridization (aCGH)

aCGH was performed as described in details (Ilic et al., 2012).

## Author disclosure statement

There are no competing financial interests in this study.

## Figures and Tables

**Fig. 1:**
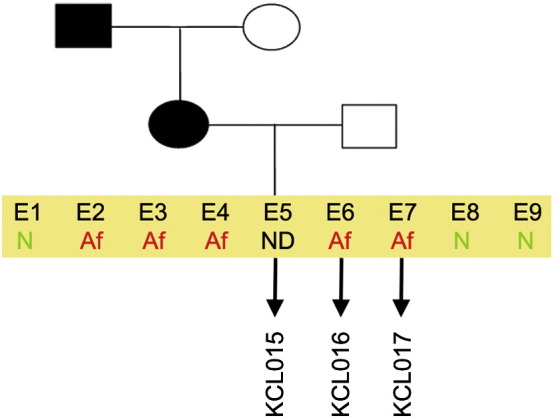
Genetic pedigree tree of the couple undergoing PGD for the VHL syndrome.

**Fig. 2:**
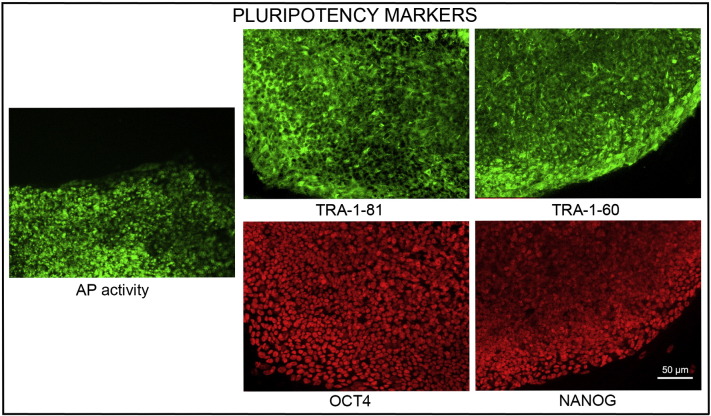
Expression of pluripotency markers. Pluripotency is confirmed by immunostaining (Oct4, Nanog, TRA-1-60, TRA-1-81) and alkaline phosphatase (AP) activity assay. Actin stress fibers, visualized with rhodamine-phalloidin (red), are present in both feeders and hES cell colonies, whereas AP activity (green) is detected only in hES cells. Scale bar, 50 μm.

**Fig. 3:**
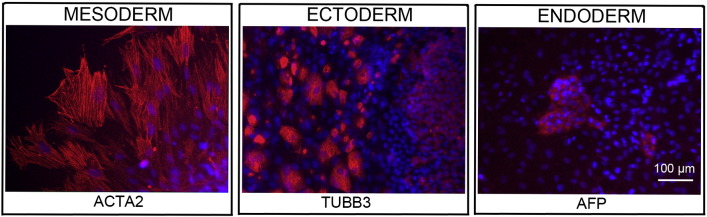
Differentiation of three germ layers in vitro is confirmed by detection of markers: smooth muscle actin (ACTA2, red) for mesoderm, β-III tubulin (TUBB3, red) for ectoderm and α-fetoprotein (AFP, red) for endoderm. Nuclei are visualized with Hoechst 33,342 (blue). Scale bar, 100 μm.

**Table 1:** Genotyping. Microsatellite markers specific for chromosomes 13, 18, 21, X and Y were amplified. The allele sizes in bp for markers on chromosomes 13, 18, and 21 are listed in the table.

Chr	Marker	Allele 1	Allele 2
13	D13S252	295	295
D13S305	450	454
D13S325	281	290
D13S628	450	457
D13S634	401	408
18	D18S386	363	378
D18S390	372	372
D18S391	214	218
D18S535	478	482
D18S819	412	416
D18S976	480	483
D18S978	211	220
21	D21S11	245	249
D21S1409	213	225
D21S1411	313	321
D21S1435	185	185
D21S1437	331	331
